# Rebozo and advanced maternal postures: A promising set of intrapartum interventions to reduce persistent occiput posterior position of the fetal head

**DOI:** 10.18332/ejm/191511

**Published:** 2024-10-18

**Authors:** Simona Fumagalli, Laura Antolini, Antonella Nespoli, Maria Panzeri, Teresa Terenghi, Simona Ferrini, Roberta Spandrio, Isabella Marzia Maini, Anna Locatelli, Sara Ornaghi

**Affiliations:** 1 University of Milan-Bicocca, School of Medicine and Surgery, Monza, Italy; 2 Unit of Obstetrics, Foundation IRCCS San Gerardo dei Tintori, Monza, Italy

**Keywords:** occiput posterior position, maternal postures, Rebozo technique, Spinning Babies® procedures, persistent occiput posterior position

## Abstract

**INTRODUCTION:**

Spinning Babies® procedures and the Rebozo technique have been recently implemented as additional interventions in laboring women with a fetus in occiput posterior position (OPP) to favor the rotation to an anterior position, which improve birth experience and health outcomes. Our study aimed to compare the probability of occurrence of persistent OPP (POPP) of the fetal head at the second stage of labor between retrospective and prospective cohorts and to assess associated sociodemographic, obstetric and intrapartum factors.

**METHODS:**

We conducted a combined prospective and retrospective cohort study including 1500 women giving birth in 2017 (retrospective cohort) and 779 between 15 May and 15 December 2023 (prospective cohort). Each cohort was divided into two sub-cohorts depending on presence of OPP. Primary outcomes were compared the probability of occurrence of POPP in the two OPP sub-cohorts by a log binomial regression and logistic regression. A p<0.05 was considered statistically significant. Data analysis was performed using Stata/MP18.0

**RESULTS:**

The proportion of OPP at the onset of labor was similar between the two cohorts (34.9% vs 35.1%). The probability of occurrence of POPP was significantly lower in the prospective OPP sub-cohort (27.7%, n=65/235) compared to the retrospective OPP sub-cohort (35.8%, n=154/430) (risk difference, RD= -0.081; 95% CI: -0.15 – -0.008; p=0.031). In the retrospective OPP sub-cohort, maternal age ≥35 years (RD=0.096; 95% CI: 0.001–0.190, p=0.044) and nulliparity (RD= -0.100; 95% CI: -0.190 – -0.001, p=0.036) were significantly associated with the probability of POPP.

**CONCLUSIONS:**

Our findings suggest a potential benefit of a set of interventions combining Spinning Babies® and the Rebozo technique in decreasing the probability of POPP.

## INTRODUCTION

Fetal occiput posterior position (OPP), identified when the back of the fetal head lies towards the mother’s back, is the most common ‘fetal malposition’, and occurs in approximately 30–40% of women at the onset of the active phase of labour^1^.The rate of a persistent occiput posterior position (POP) is 20–30% at 10 cm dilatation, and 5–13% at birth^2^. Several factors have been associated with OPP during labor, including advanced maternal age^3^, high maternal body mass index (BMI, kg/m^2^)^4^, loose abdomen, and nulliparity^1^. Women of African-American ethnicity, with a narrow pubic arch angle typical of the anthropoid and android pelvis^5,6^ are also more likely to experience OPP. In addition, anterior positioned placenta, fetal macrosomia, late- and post-term pregnancy, epidural analgesia, and untimely artificial rupture of membranes have been recognized as antenatal and intrapartum factors associated with a higher probability of OPP^7-9^.

The decrease of OPP rate throughout labor until birth is due to spontaneous rotation to a more favorable occiput anterior position (OAP), usually just before the start or at the beginning of the second stage of labour^10-12^. About 10–20% of women with a fetus in OPP in the early second stage of labor will not experience an anterior rotation of the fetal head before birth^1^, and this associates with a lower probability to experience a spontaneous vaginal birth^2,3,13^, while increasing the risk of both maternal and neonatal adverse^14,15^. It exposes to prolonged labor, instrumental delivery, increased cesarean section rates, high-degree perineal lacerations, and postpartum hemorrhage. Also, POPP has been related to low Apgar scores and cord blood pH values at birth, admission to neonatal intensive care unit, and neonatal encephalopathy^3,9,14,16^.

Use of maternal postures may facilitate spontaneous fetal rotation of OPP to the more favorable OAP^17^, referred to as ‘optimal fetal positioning’ and associated with better maternal and neonatal health outcomes. Maternal postures, which include hands-and-knees and lateral recumbent position or Sims position (semi-prone) as it is widely called in midwifery practice^18^, seek to non-invasively promote flexion of the fetal head to favor its spontaneous rotation into the OAP^17^. There is limited evidence on the appropriate maternal postures to use, the optimal time to adopt the postures, or their effectiveness^1,19-27^. More recently, additional interventions have been proposed as non-invasive alternatives to maternal postures for favoring anterior rotation of an OP fetus: the Spinning Babies® procedures and the Rebozo technique^28,29^. The Spinning Babies® procedures, developed by a homebirth midwife from Minnesota, USA^30^, focus on the importance of the role of soft tissues, including ligaments, muscles, and connective tissue, in fetal head’s correct positioning (i.e. anterior) and labor onset and progress. It comprises advanced maternal postures, such as forward-leaning inversion and side-lying release, and manipulations to favor sacral mobility and uterine fascial release. Briefly, the forward-leaning inversion uses stretch receptors to untwist and lengthen the uterosacral ligament for increased maternal comfort, dilation ease, and improved fetal position, whereas the side-lying release allows uterus repositioning, pelvic floor softening, and buttock and hip muscles release^28^. The Rebozo technique is based on the use of the rebozo (a woven shawl) to massage or shift the woman’s pelvis or uterus, therefore encouraging fetal rotation and optimum positioning, as well as promoting maternal comfort. It is a long-standing traditional practice from central and southern Mexico, which, in recent years, has spread in other countries, including the United States, Denmark, and Italy^29,31^. By acting on uterus repositioning, pelvic floor muscle and uterine ligaments and fascia release, the supposed mechanisms of action underlying both the Spinning Babies® procedures and the Rebozo technique are similar. Such mechanisms can possibly create the most favorable conditions to allow anterior rotation of the OP fetal head^28,29,32^.

The widespread knowledge and awareness of the potential benefits of these practices have increased the interest of midwives employed at IRCCS San Gerardo Hospital - Monza, Italy, in their use, thus leading to training sessions for both the Spinning Babies® procedures and the Rebozo technique. Subsequently, midwives’ knowledge and operational confidence in both interventions has progressively increased, resulting in the gradual introduction of such interventions into routine clinical practice starting from 2022. In Italy, maternity care is part of the public service provided by the National Health System which offers free universal health coverage funded by taxation. Births mainly take place in Obstetrics units, which host both low and high-risk women. Midwives play a crucial role in these units, providing one-to-one care throughout labor and birth for all women. They are responsible for assisting low-risk vaginal births, ensuring that women receive continuous support and professional care during the birthing process.

Several randomized clinical trials investigating maternal postures for increasing the rate of OAP in laboring women with an OP fetus have been completed in the last twenty years^1,19-27^, with most studies failing to demonstrate the effectiveness of these interventions^19-24^. However, these studies show substantial limitations, including a clinical instead of a sonographic evaluation of OPP^24^, the maintenance of the investigated maternal posture for a short period of time (≤30 minutes)^19,23,24^, and an inadequate sample size^19^. In addition, in all but one trial, specifically the study by Collalecci et al.^18,20-24^, only one specific maternal posture represented the intervention under investigation. Of note, some of these trials have shown an improvement in maternal back pain and comfort associated with postures^19,23^. Considering this, and the fact that maternal postures are safe, simple to execute, non-invasive, and widely accepted by women, they are a cornerstone of the current midwifery practice in assisting laboring women with an OP fetus. Neither the Spinning Babies® procedures nor the Rebozo technique have been shown to harm the mother or the fetus^28,29,32^. Although being non-invasive and safe, neither intervention has been rigorously evaluated in a research study as to its effectiveness in determining anterior rotation of an OP fetus.

Our study aimed to compare the probability of occurrence of POPP of the fetal head at the second stage of labor between women who gave birth in 2017 (retrospective cohort) and between 15 May and 15 December 2023 (prospective cohort) at the IRCCS San Gerardo Hospital maternity-care center. The secondary aim was to describe sociodemographic, obstetric, and intrapartum factors which have an impact on the probability of occurrence of POPP of the fetal head within the retrospective OPP sub-cohort.

## METHODS

### Study design and setting

This was a combined prospective and retrospective cohort study including women giving birth in 2017 (retrospective cohort) and between 15 May and 15 November 2023 (prospective cohort), at IRCCS (Istituto di Ricovero e Cura a Carattere Scientifico) San Gerardo Hospital- Monza (Italy), a tertiary-care center.

The research site was an academic Obstetrics Unit of a maternity hospital in Northern Italy with approximately 2500 births/year. The Obstetrics Unit hosts both low and high-risk women and offers one-to-one midwifery care throughout labor and birth to all women. Women are admitted to the maternity unit in the active phase of the first stage of labor, defined as regular, painful, and strong contraction with cervical dilatation ≥4 cm. The evaluation of the fetal head position was performed clinically by Leopold’s manoeuvres and vaginal examination. According to the local protocol the adoption of mobility and an upright position during labor is promoted.

*Retrospective cohort (1 January to 31 December 2017)*

Only maternal postures, including upright, semi-recumbent, lateral recumbent, and hands-and-knees, have been commonly used by midwives assisting women in the first stage of labor with OP fetuses.

*Prospective cohort (15 May to 15 November 2023)*

In the last couple of years, training sessions for both the Spinning Babies® procedures and the Rebozo technique have been undertaken by midwives to improve their knowledge and operational confidence in both interventions, which, in turn, have been progressively introduced into clinical practice. However, their use in laboring women with an OP fetus has been inconsistent.

### Participants and data collection

Women who were aged ≥18 years, in active phase of the first stage of labor, with a singleton term fetus (≥37 0/7 weeks of gestation) were included in the analyses. Women without a clinical diagnosis of fetal head position before 8 cm of cervical dilation were excluded. The participants were selected using total population sampling, ensuring all eligible women within the study period and location were included.

For the definition of the retrospective cohort, records of women who gave birth in the study period (from 1 January to 31 December 2017) were retrospectively screened within the electronic birth register to identify eligible women.

**Figure 1:**
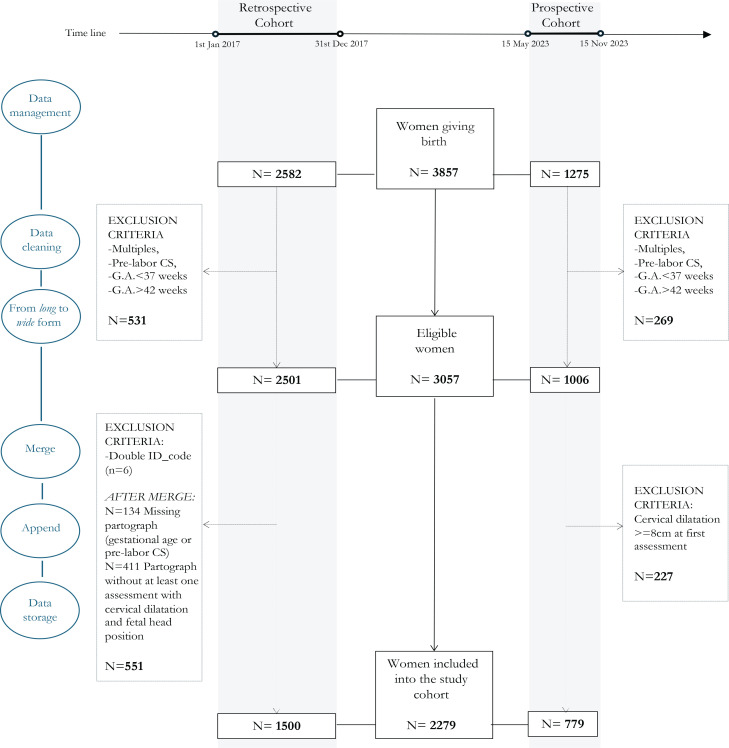
Description of the selection process to identify the final study cohort

Data sources were two different electronic databases that record data from medical charts. A data cleaning process was performed to ensure accuracy, consistency and completeness of data before final data storage (Figure 1). A systematic data cleaning and monitoring for the outlier, missing and improbable values was checked. Data indicating adherence to study inclusion and exclusion criteria were subjected to edit checks, as well as the protocol-specified parameters such as the presence of at least one vaginal examination before 8 cm of cervical dilation with a description of fetal head position. Finally, to ensure consistency, data formats and values were standardized and normalized.

For the definition of the prospective cohort, data of all eligible women who gave birth in the study period (from 15 May to 15 November 2023) with a clinical diagnosis of OPP of fetal head before 8 cm of cervical dilation, were prospectively collected from the medical charts and recorded in a database by a research assistant. Systematic data cleaning and monitoring for the outlier, missing and improbable values was performed.

The prospective database was merged into the retrospective database adding new observations of prospective cohort to existing variables. Finally, data formats and values were standardized and normalized to ensure consistency. Patient privacy was ensured by coding and anonymous processing by investigators, which was uploaded to an online data platform. After data cleaning and quality checks, the database was kept secure in a locked and encrypted online database system. Each cohort (retrospective and prospective) were divided into two sub-cohorts depending on the presence/absence of OPP.

### Variables

The primary outcomes were defined as the probability of occurrence of occiput posterior position (OPP) of the fetal head at the first stage of labor and the probability of occurrence of persistent occiput posterior position (POPP) of the fetal head at the second stage of labor.

To identify the fetal head position at the first stage of labor, we considered the first evaluation of the fetal head position clinically diagnosed between 3 and 8 cm of cervical dilation.

Secondary outcomes included OPP of fetal head at birth and modes of birth (spontaneous vaginal birth, vacuum assisted vaginal birth, and cesarean section - CS).

To identify factors which may have an impact on the probability of occurrence of POPP, the following predictor variables were extracted from the data sources: sociodemographic characteristics (maternal age at birth, ethnicity, BMI); obstetric factors (parity, gestational age at birth, previous CS, healthy pregnancy, premature rupture of membranes and onset of labor) and intrapartum interventions (epidural analgesia and the use of oxytocin in labor). All the listed variables may reasonably have an impact on the probability of occurrence of POPP within each cohort, without impact on the probability of belonging to each specific cohort (which could generate the presence of confounder). This is motivated by the two timeframe which were contiguous and consist both in a few months’ time-windows. Of the listed variables, only parity is considered as a possible effect modifier since the length of the active phase of first stage of labor is expected to be greater in nulliparous, and this could impact on the probability of occurrence of POPP.

Parity was dichotomized into ‘nulliparous’ versus ‘multiparous’, gestational age was calculated in weeks and dichotomized according to the classification of term-pregnancy^33^ into ‘early- and full-term’ (37 0/7 weeks of gestation through 40 6/7 weeks of gestation) versus ‘late- and post-term’ (41 0/7 weeks of gestation through 42 0/7 weeks of gestation), BMI was dichotomized into ‘≥30 kg/m^2^’ and ‘<30 kg/m^2^’, ethnicity was dichotomized into ‘Caucasian’ versus ‘not Caucasian’.

### Statistical analysis

Descriptive statistics of the baseline characteristics were calculated overall and according to study sub-cohorts. For continuous variables, their distributions were visually assessed to ensure they were approximately normally distributed. Means and standard deviations were calculated, and Student’s t-test was used to determine if there was a significant difference in the means of these continuous variables between the groups. For categorical variables, frequencies and percentages were calculated, and the chi-squared test was used to assess differences between groups.

We compared primary outcomes between women giving birth in the retrospective and prospective OPP sub-cohorts using a logistic regression model, treating the retrospective sub-cohort as the reference group. From the logistic regression model, we calculated the predicted probabilities of the outcome for each group and derived the risk difference (RD) and its corresponding 95% confidence interval (95% CI). The model estimated the relative risk (RR) of POPP associated with the intervention period, nulliparity and their interaction. Additionally, the risk difference (RD) and its corresponding 95% confidence interval (95% CI) were calculated from the predicted probabilities of the outcome for each group.

The association between sociodemographic, obstetric, and intrapartum factors and the probability of occurrence of POPP of the fetal head at the second stage in the retrospective cohort was estimated using the log-binomial regression model to define the RD and its corresponding 95% CI. All tests performed were two-sided and a p<0.05 considered statistically significant.

Analyses was performed with the STATA software v. 16 (College Station, TX: StataCorp LLC).

### Ethical considerations

The study was performed in line with the principles of the Declaration of Helsinki. Approval was granted by the Institutional Review Board of the University of Milan-Bicocca, Italy (protocol No. 106, 14 October 2015). At our Hospital, women provide a written consent to the use of their clinical anonymized and deidentified data upon admission.

## RESULTS

During the study period, 3857 women gave birth, 2582 in the retrospective cohort and 1275 in the prospective cohort. Of these, 3057 (79.2%) met the inclusion criteria and were considered eligible. In the retrospective cohort, 551 women (26.8%) were excluded after the data cleaning process, while in the prospective cohort 227 (22.5%) women were excluded for a clinical diagnosis of fetal head position performed after 8 cm of cervical dilation (Figure 1).

**Figure 2:**
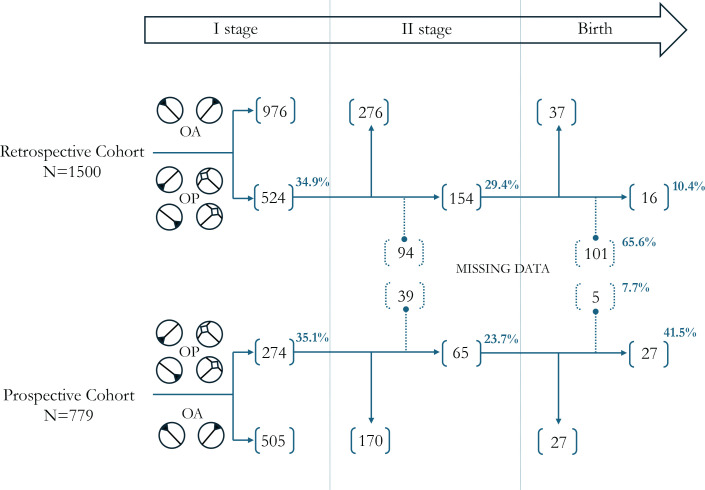
Distribution of women according to fetal head position during labor

A total of 1500 women were included in the retrospective cohort, of these 524 (34.9%, 95% CI: 0.32–0.37) had an OPP at the first stage of labor. In the prospective study cohort, a total of 779 women were included, and 274 of them (35.2%, 95% CI: 0.32–0.38) had an OPP of the fetal head at the first stage of labor (Figure 2).

Sociodemographic and obstetric characteristics of women who gave birth in the retrospective cohort are described in Table 1. Data are summarized for the entire study cohort and according to OP status. In the retrospective cohort, the average age of mothers was 32.1 years (SD=5.2), and Caucasian women (p=0.021) and women aged ≥35 years (p=0.032) were more likely to have the fetal head in OPP during the first stage of labor. The 55.9% of the women were nulliparous (n=839), 79.7 % had a uncomplicated pregnancy without chronic diseases (n=1196) and a spontaneous onset of labor was reported in 71.7% of cases. We found no significant differences regarding all the obstetric characteristics considered between sub-cohorts (Table 1).

**Table 1:** General and obstetric characteristics of women of the retrospective cohort according to the position of fetal head at the first stage of labor

*Characteristics*	*Overall (N=1500)*		*OPP (N=524)*		*OAP (N=976)*		*p*
**General**							
	***Mean***	***SD***	***Mean***	***SD***	***Mean***	***SD***	
Maternal age (years)	32.1	5.2	32.4	5.3	31.9	5.2	0.076
BMI pre-conception	22.5	3.8	22.5	3.8	22.5	3.9	0.879
Gestational weight gain	12.8	4.6	13.0	4.6	12.6	4.5	0.153
	***n***	***%***	***n***	***%***	***n***	***%***	
Maternal age ≥35 years	502	33.5	194	37.0	308	31.6	0.032
BMI >30	235	15.7	85	16.2	150	15.4	0.665
Ethnicity (Caucasian)	1236	82.4	448	85.5	788	80.7	0.021
**Obstetric**							
Nulliparous	839	55.9	296	56.5	543	55.6	0.751
Previous CS	142	9.5	45	8.6	97	9.9	0.394
Uncomplicated pregnancy	1196	79.73	423	80.7	773	79.2	0.484
	***Mean***	***SD***	***Mean***	***SD***	***Mean***	***SD***	
Gestational age (weeks)	39.7	1.2	39.75	1.18	39.68	1.22	0.299
	***n***	***%***	***n***	***%***	***n***	***%***	
Gestational age >40 weeks	429	28.6	152	29.0	277	28.4	0.798
PROM	643	42.9	218	41.6	425	43.6	0.469
Spontaneous onset of labor	1076	71.7	381	72.7	695	71.2	0.538

OPP: occiput posterior position. OAP: occiput anterior position. CS: cesarean section. PROM: premature rupture of membranes. Uncomplicated pregnancy is defined as a pregnancy in a woman with no chronic conditions or gestational complications. BMI: body mass index (kg/m^2^).

Considering the comparison between women with an OPP at the first stage of labor in the retrospective sub-cohort and prospective OPP sub-cohort, our results show a higher probability to have an uncomplicated pregnancy without chronic diseases (80.7% vs 51.8%, p<0.001) and a late- or post-term pregnancy (29.0% vs 20.7%, p=0.006) in the retrospective versus prospective OPP sub-cohorts. Moreover, in the prospective sub-cohort, women had a higher probability of receiving epidural analgesia compared to the retrospective sub-cohort (47.8% vs 33.6%, p<0.001) (Table 2).

**Table 2:** General and obstetric characteristics and intrapartum care of women with an OPP of fetal head at first stage of labor according to retrospective and prospective OPP sub-cohorts

	*Overall OPP sub-cohorts (N=798)*		*Retrospective OPP sub-cohort (N=524)*		*Prospective OPP sub-cohort (N=274)*		*p*
**General characteristics**							
	***Mean***	***SD***	***Mean***	***SD***	***Mean***	***SD***	
Maternal age (years)	32.3	5.2	32.4	5.3	32.2	4.8	0.483
	***n***	***%***	***n***	***%***	***n***	***%***	
Maternal age ≥35 years	232	34.9	160	37.2	72	30.6	0.089
Ethnicity (not Caucasian)	116	14.5	76	14.5	40	14.6	0.971
**Obstetric characteristics**							
Nulliparous	469	58.8	296	56.5	173	63.1	0.070
Previous CS	59	7.4	45	8.6	14	5.1	0.075
Uncomplicated pregnancy	565	70.8	423	80.7	142	51.8	<0.001
	***Mean***	***SD***	***Mean***	***SD***	***Mean***	***SD***	
Gestational age (weeks)	39.6	1.2	39.7	1.2	39.4	1.2	<0.001
	***n***	***%***	***n***	***%***	***n***	***%***	
Late/post-term pregnancy	207	25.9	152	29.0	55	20.7	0.006
Spontaneous onset of labor	576	72.2	381	72.7	195	71.2	0.644
**Intrapartum care**							
Epidural analgesia	307	38.5	176	33.6	131	47.8	<0.001
OX in labor	102	12.8	61	11.6	41	15.0	0.182

OPP: occiput posterior position. CS: cesarean section. OX: oxytocin. Uncomplicated pregnancy is defined as a pregnancy in a woman with no chronic conditions or gestational complications. Late/post-term pregnancy is defined as a gestational age between 41 0/7 weeks and 42 0/7 weeks of gestation.

**Figure 3:**
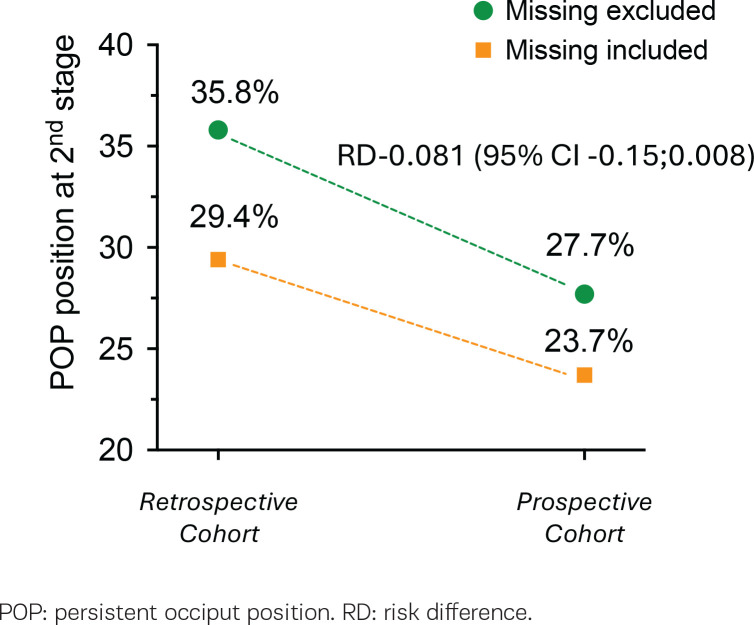
Risk difference of POPP between retrospective and prospective cohorts

The probability of occurrence of POPP at the second stage of labor was 29.4% (n=154/524) in the retrospective sub-cohort and 23.7% (n=65/274) in the prospective subcohort. The data on the fetal head position at second stage of labor was not available (missing) for a total of 133 women (16.6%), 94 were in the retrospective sub-cohort and 39 in the prospective sub-cohort. After removing missing data, the probability of occurrence of POPP was 35.8% in the prospective intervention period sub-cohort, with a significant reduction of the probability in the prospective sub-cohort (RD= -0.081; 95% CI: -0.15 – -0.008; p=0.031). Figure 3 shows the RD of POPP among sub-cohorts according to exclusion or inclusion of missing data. The multivariate log-binomial regression model showed that prospective cohort and nulliparity had a significant impact on the probability of occurrence of POPP with a borderline interaction between the ‘cohort’ (Table 3).

**Table 3:** Results of log-binomial regression model for predictors of POPP of fetal head at second stage of labor

	*Risk of POPP %*	*RD*	*95% CI*	*p*
Prospective cohort	35.8	-0.081	-0.15 – -0.008	0.029
Prospective cohort	42.1	-0.100	-0.19 – -0.005	0.038
Nulliparous	32.1	-0.171	-0.29 – -0.05	0.007
Interaction	28.9	0.139	-0.01–0.29	0.074

POP: persistent occiput position. RD: risk difference.

The association between sociodemographic, obstetric and intrapartum factors and the probability of occurrence of POPP at the second stage in the retrospective OPP sub-cohort are described in Table 4. The log-binomial regression model, performed on the retrospective OPP sub-cohort (Table 5), identified maternal age ≥35 years and nulliparity, the only two maternal characteristics significantly associated with the probability of having fetal head rotation to an OAP at the second stage of labor.

**Table 4:** General and obstetric characteristics and intrapartum care of women of the retrospective OP subcohort according to the position of fetal head at the second stage of labor

*Characteristics*	*POPP at stage II (N=154)*		*No POPP at stage II (N=276)*		*p*
	*n*	*%*	*n*	*%*	
**General**					
Maternal age ≥35 years	67	43.5	93	33.7	0.044
BMI >30	29	18.8	40	14.5	0.240
Ethnicity (Caucasian)	128	83.1	245	88.8	0.098
**Obstetric**					
Nulliparous	87	56.5	184	66.7	0.036
Previous CS	8	5.2	29	10.5	0.065
Uncomplicated pregnancy	127	82.5	220	79.7	0.487
Gestational age (late/post-term)	43	27.9	84	30.4	0.584
PROM	66	44.2	117	42.4	0.723
Spontaneous onset of labor	111	72.1	197	71.4	0.877
Epidural analgesia	56	36.4	108	39.1	0.571
OX in labor	20	13.0	30	10.9	0.511

POPP: persistent occiput posterior position. BMI: body mass index (kg/m^2^). CS: cesarean section. PROM: premature rupture of membranes. OX: oxytocin.

Uncomplicated pregnancy is defined as a pregnancy in a woman with no chronic conditions or gestational complications. Late/post-term pregnancy is defined as a gestational age between 41 0/7 weeks and 42 0/7 weeks of gestation.

**Table 5:** Results of log-binomial regression model for predictors of POPP of fetal head at second stage of labor in retrospective OPP sub-cohort (N=430 observations)

*Variable (yes/no)*	*Risk of POPP baseline group %*	*RD*	*95% CI*	*p*
Maternal age ≥35 years	32.2	0.096	0.00–0.19	0.046
BMI >30	34.6	0.074	-0.05–0.20	0.251
Caucasian ethnicity	45.6	-0.112	-0.25–0.02	0.109
Nulliparous	42.1	-0.100	-0.19 – -0.005	0.038
Previous CS	37.1	-0.155	-0.29 – -0.01	0.065
Uncomplicated pregnancy	32.5	0.041	-0.07–0.15	0.480
Late/post-term pregnancy	36.6	-0.027	-0.13–0.07	0.581
PROM	35.1	0.016	-0.07–0.11	0.723
Spontaneous onset of labor	36.0	-0.007	-0.11–0.09	0.877
Epidural analgesia	36.8	-0.026	-0.12–0.06	0.569
OX in labor	35.3	0.047	-0.09–0.19	0.519

RD: risk difference. POPP: persistent occiput posterior position. BMI: body mass index (kg/m^2^). CS: cesarean section. PROM: premature rupture of membranes. OX: oxytocin. Uncomplicated pregnancy is defined as a pregnancy in a woman with no chronic conditions or gestational complications. Late/post-term pregnancy is defined as a gestational age between 41 0/7 weeks and 42 0/7 weeks of gestation.

Women aged ≥35 years had a higher probability of occurrence of POPP (RD=0.096; 95% CI: 0.001–0.19; p=0.046) while being nulliparous was associated with a reduction of POPP probability (RD= -0.100; 95% CI: -0.19 – -0.005; p=0.038). History of CS showed a trend towards decreased probability of occurrence of POPP, although not reaching statistical significance (RD= -0.155; 95% CI: -0.290 – -0.010; p=0.065) (Table 5).

Considering the probability of occurrence of POPP at birth according to fetal head position at second stage of labor, Figure 2 shows the difference in the proportion of missing data within retrospective and prospective sub-cohort (65.6% vs 7.7%). The high proportion of missing data relating to the fetal position at birth (65.6%) in the retrospective sub-cohort does not allow comparison between OPP sub-groups.

Having POPP of fetal head at second stage of labor influences mode of birth with a lower risk (probability) to experience a spontaneous vaginal birth, both in the retrospective (83.1% vs 89.9%, p=0.043) and in the prospective (64.6% vs 92.9%, p<0.001) OPP sub-cohorts. Consistently, the probability of having a vacuum-assisted vaginal birth or a CS, increased in women with POPP in both cohorts. However, this difference was statistically significant only in the prospective cohort (9.2% vs 2.4%, p=0.019; 26.2% vs 4.7%, p<0.001).

## DISCUSSION

The findings of this preliminary combined prospective and retrospective cohort study show that after the introduction of the Spinning Babies® procedures and the Rebozo technique into clinical practice (prospective cohort), the probability of occurrence of POPP at second stage substantially decreased.

The proportion of women with an OPP of fetal head at the onset of labor is similar within cohorts (34.9% and 35.2%) and is consistent with previous studies reporting a 30–40% OPP probability in early active labour^1,2^. Women with an OPP were more frequently aged ≥35 years and Caucasian. Maternal age and nulliparity were the two characteristics significantly associated with the probability of occurrence of POPP at second stage of labor. Regardless of study cohort, having POPP of fetal head at second stage was a risk factor for CS.

Several maternal characteristics have been recognized to be associated with increased odds of OPP, including advanced maternal age, increased maternal body mass index and African-American ethnicity^3-6,14,34^. Our findings show an association between maternal aged ≥35 years and OPP, from the onset of active labor to the start of second stage. Whereas past researchers have found an association between nulliparity and fetal malposition^3^, the present study has shown a significant increase of the probability to have a fetal head rotation in OAP before the second stage of labor in nulliparous women.

Although evidence is lacking on the appropriateness, timing, and effectiveness of maternal postures for increasing the rate of OAP in laboring women with an OP fetus^1,22,35,36^, knowledge on the pathways by which maternal posture might work to rotate the baby anteriorly is consistent^22,37^. It is likely that fetal head flexion and anterior rotation are induced by changes in pelvic angles and diameters combined with the force of gravity and buoyancy. The supposed mechanisms of action underlying both the Spinning Babies® procedures and the Rebozo technique are similar. These mechanisms include uterus repositioning, pelvic floor muscle and uterine ligaments and fascia release, which can possibly create the most favorable conditions to allow anterior rotation of the OP fetal head^28,29,31,32^. This hypothesis is further supported by our finding of a positive impact of the introduction of the Spinning Babies® procedures and the Rebozo technique into clinical practice. The comparison between the two OPP sub-cohorts (retrospective and prospective) showed a reduction of the probability to have a POPP during the period when the Spinning Babies® procedures (i.e. forwardleaning inversion and side-lying release positions) and/or Rebozo technique were offered to women with an OPP at the onset of labor.

Considering the period when only maternal postures, including upright, semi-recumbent, lateral recumbent, and hands-and-knees, were used, we observed a probability of occurrence of POPP in line with findings from previous trials on maternal postures in fetal malposition^21,22^. Studies have demonstrated that women who have an OPP of fetal head in the early second stage of labor have a higher risk to undergo CS, because spontaneous rotations are unlikely once the second stage has begun^3,13^. Our findings are consistent with this finding, reporting an impact on mode of birth with a lower risk (probability) to experience a spontaneous vaginal birth in women having POPP of fetal head at second stage of labor.

Our results support the implementation into clinical practice of Spinning Babies® procedures and Rebozo technique, which should be considered as non-invasive and safer alternatives to maternal postures for favoring anterior rotation of an OP fetus. A systematic and consistent registration of these procedures in medical charts is pivotal for monitoring the adoption and ensure clinical audit. Neither the Spinning Babies® procedures nor the Rebozo technique has been rigorously evaluated in a research study as to its effectiveness in determining anterior rotation of an OP fetus.

### Strengths and limitations

The strengths of our study include a large sample size that included women with broadly similar characteristics. This study is not without limitations. First, the retrospective nature of the design prohibits us from removing the tendency of confounding and establishing the causality of the association. Second, it is possible that this study was underpowered to assess differences in secondary outcomes, including fetal position at birth, due to the higher proportion of missing data. Third, the lack of follow-up data on study participants is an important limitation and a potential source of bias. Our findings are from a single center, and may not be generalizable to different settings. Finally, we did not measure maternal birth satisfaction at birth and other medium- and long-term outcomes.

## CONCLUSIONS

Our study highlights a lower probability of occurrence of POPP of the fetal head at the second stage of labor in women who gave birth when the Spinning Babies® procedures and/ or Rebozo technique were offered, compared to those who did not receive these procedures. These results suggest a positive influence of Spinning Babies® procedures and/or Rebozo technique on the fetal head rotation. Given these findings, there is a strong suggestion for further research to explore the effects of a set of these non-invasive interventions. Specifically, conducting a randomized clinical trial would be essential to confirm the benefits observed and to establish a clear causal relationship. Our research contributes to the existing knowledge by identifying potential non-invasive methods to reduce the probability of occurrence of POPP, thus improving maternal and fetal outcomes during labor. Future studies should focus on larger sample sizes and diverse settings to generalize these findings and explore additional non-invasive techniques.

## Data Availability

The data supporting this research are available from the authors on reasonable request.
